# The implications of a Silurian and other thylacocephalan crustaceans for the functional morphology and systematic affinities of the group

**DOI:** 10.1186/s12862-014-0159-2

**Published:** 2014-08-22

**Authors:** Carolin Haug, Derek E G Briggs, Donald G Mikulic, Joanne Kluessendorf, Joachim T Haug

**Affiliations:** 1 Department of Biology II and GeoBio-Center, LMU Munich, Großhaderner Str. 2, Martinsried-Planegg, 82152, Germany; 2 Department of Geology and Geophysics, Yale University, New Haven, CT, USA; 3 Yale Peabody Museum of Natural History, Yale University, New Haven, 06520, CT, USA; 4 llinois State Geological Survey, Champaign, 61820, IL, USA; 5 Weis Earth Science Museum, University of Wisconsin - Fox Valley, Menasha, 54952, WI, USA

**Keywords:** Waukesha, Wisconsin, Solnhofen lithographic limestones, Predatory crustaceans, Remipedia

## Abstract

**Background:**

Thylacocephala is a group of enigmatic extinct arthropods. Here we provide a full description of the oldest unequivocal thylacocephalan, a new genus and species *Thylacares brandonensis*, which is present in the Silurian Waukesha fauna from Wisconsin, USA. We also present details of younger, Jurassic specimens, from the Solnhofen lithographic limestones, which are crucial to our interpretation of the systematic position of Thylacocephala. In the past, Thylacocephala has been interpreted as a crustacean ingroup and as closely related to various groups such as cirripeds, decapods or remipeds.

**Results:**

The Waukesha thylacocephalan, *Thylacares brandonensis* n. gen. n. sp., bears compound eyes and raptorial appendages that are relatively small compared to those of other representatives of the group. As in other thylacocephalans the large bivalved shield encloses much of the entire body. The shield lacks a marked optical notch. The eyes, which project just beyond the shield margin, appear to be stalked. Head appendages, which may represent antennulae, antennae and mandibles, appear to be present. The trunk is comprised of up to 22 segments. New details observed on thylacocephalans from the Jurassic Solnhofen lithographic limestones include antennulae and antennae of *Mayrocaris bucculata*, and endites on the raptorial appendages and an elongate last trunk appendage in *Clausocaris lithographica*. Preserved features of the internal morphology in *C. lithographica* include the muscles of the raptorial appendage and trunk.

**Conclusions:**

Our results indicate that some ‘typical’ thylacocephalan characters are unique to the group; these autapomorphies contribute to the difficulty of determining thylacocephalan affinities. While the new features reported here are consistent with a eucrustacean affinity, most previous hypotheses for the position of Thylacocephala within Eucrustacea (as Stomatopoda, Thecostraca or Decapoda) are shown to be unlikely. A sister group relationship to Remipedia appears compatible with the observed features of Thylacocephala but more fossil evidence is required to test this assertion. The raptorial appendages of Thylacocephala most likely projected 45 degrees abaxially instead of directly forward as previously reconstructed. The overall morphology of thylacocephalans supports a predatory mode of life.

## Background

The monophyletic group Thylacocephala is known to range from at least 435 million years ago (Silurian) [1],[2] to 84 million years ago (Cretaceous) [3]. Vannier et al. [4] described a possible Cambrian species, *Zhenghecaris shankouensis*, from the lower Cambrian Chengjiang fauna of China and discussed whether other Cambrian arthropod species (of *Isoxys* and *Tuzoia*) might represent thylacocephalans (see also [5]). These arthropods, however, do not preserve the characteristic raptorial appendages [4],[6]. The enigmatic *Ainiktozoon loganense*, from the Lower Silurian of Lesmahagow, Scotland, which has compound eyes and possible spiny limbs, has also been interpreted as a thylacocephalan [7]. Its complex morphology [8], however, is not easy to reconcile with that of thylacocephalans. Thylacocephalans are characterised by a large bivalved shield often termed ‘carapace’ that encloses almost the entire body. Many representatives are known only from their valves. Where other aspects of the ‘soft part’ morphology are preserved thylacocephalans typically show a pair of large anterior compound eyes and three pairs of large sub-chelate raptorial appendages. It is not clear to which body segments these raptorial appendages belong. Posterior of them the trunk consists of a series of homonomous segments that bear relatively simple appendages.

The interpretation of the morphology and systematic position of Thylacocephala has been controversial since their discovery [4],[6],[9]-[12]. The first specimens of Thylacocephala described were isolated shields of *Concavicaris sinuata* interpreted at that time (1868) as phyllocarids [13]. In the 1880s thylacocephalans from the Cretaceous of Sahel Alma, Lebanon, were interpreted as larvae of stomatopod crustaceans [14],[15], see [3]. A century later species from the Jurassic of Osteno, Italy, were interpreted as relatives of thecostracan crustaceans, i.e. barnacles and their parasitic relatives [16]. Other thylacocephalan species have been compared to non-stomatopod malacostracan crustaceans, particularly decapods [4],[12],[17]. Thus, while there is some agreement that thylacocephalans are representatives of Eucrustacea, their placement within this higher taxon is uncertain.

The best preserved specimens are from Jurassic Lagerstätten. Details of the internal morphology are known from the famous La Voulte Lagerstätte in south-eastern France [12],[18]. Specimens from the Jurassic of southern Germany preserve possible appendages anterior of the raptorial limbs [19]. Even these details, however, have not resulted in a more satisfactory systematic assignment, possibly because these relatively young species are derived representatives of the group. The evidence of these Jurassic fossils has led to a consensus on the mode of life of Thylacocephala, which are thought to have been mobile predators or ambush predators [4],[20].

The oldest well preserved material of unequivocal thylacocephalans is from the Silurian Waukesha biota of Wisconsin, USA. It is to these Paleozoic fossils that we look for evidence of the more plesiomorphic morphology of the group, and possible insights into the systematic affinities of Thylacocephala. New details of Jurassic species from the Solnhofen lithographic limestones also provide evidence of the possible systematic affinity and ecology of Thylacocephala.

## Methods

### Material

#### The Silurian specimens

UWGM 1748–1750, 1767–1769 (all with part and counterpart) are held by the Geology Museum, Department of Geology and Geophysics, University of Wisconsin, Madison, U.S.A.. These six specimens are from the Brandon Bridge Formation (late Telychian) at Waukesha, near Milwaukee, Wisconsin (see [21]-[23] for details of the setting). The enclosing lithology is finely laminated organic-rich argillaceous mudstone and dolomudstone that occurs in the lowest 2 m of the Brandon Bridge strata. Fine scale lamination with limited bioturbation suggests deposition in an anoxic, possibly brackish, environment. Arthropods, dominantly represented by exuviae, are the major component of the fauna and include trilobites, phyllocarids and ostracods, and a number of undescribed arthropods and worm-like animals of uncertain affinity [1],[2]. Shelly fossils are rare and usually decalcified. The exceptionally preserved assemblage clearly represents an unusual environmental setting related to restricted circulation associated with initial flooding at the beginning of a sequence [24].

#### The Jurassic specimens

The Jurassic specimens investigated here come from the lithographic limestones of the Solnhofen area, southern Germany. Specimens are held in the collection of the Staatliches Museum für Naturkunde Stuttgart (SMNS 67901, collected by Michael Fecke, Langenberg; SMNS 70193/1–70193/5, collected by Roger Frattigiani, Laichingen). Two species are represented, *Clausocaris lithographica* (SMNS 67901, SMNS 70193/1–70193/4) and *Mayrocaris bucculata* (SMNS 70193/5). As is often the case with fossils from the Solnhofen area, for most of the specimens the locality is unknown: SMNS 67901 and SMNS 70193/3 are exceptions - both come from Eichstätt. High resolution images will be reposited in the Staatliches Museum für Naturkunde Stuttgart.

The Solnhofen Limestone is a pure laminated micritic limestone interpreted as a result of deposition in a restricted lagoon [25]. Limited circulation led to salinity-stratified water and benthic anoxia. The diverse fauna includes species of *Archaeopteryx* and *Compsognathus*, pterosaurs, fishes, shrimps and other arthropods, molluscs, echinoderms and rarer insects and plants.

#### Documentation methods

The Silurian specimens were photographed with a Canon Rebel T3i and a MPE-65 mm macrolens. Cross-polarised light was provided by Canon Macro Twin Flash MT 24. Several image details were stitched to generate a complete image of the specimens with Adobe Photoshop CS3. Resulting images were color-inverted and their histograms optimised. Prominent structures were traced by hand and color marked. Documentation of the Jurassic specimens followed the principles of fluorescence composite imaging and macro-fluorescence imaging (see [26],[27]). 3D-models were produced with Blender.

## Results

### Systematic paleontology

This published work and the nomenclatural acts it contains have been registered in Zoobank: http://zoobank.org/References/955F7A06-15DC-4118-A40B-3D773205714C.

Euarthropoda *sensu*[28]

Crustacea *sensu lato sensu*[29]

Eucrustacea *sensu*[28]

Thylacocephala *sensu*[30]

*Thylacares* gen. nov.

LSID: urn:lsid:zoobank.org:act:29D50895-5A01-4D9E-B937-5E49907BBEAE

*Derivatio nominis: Thylax* (Gr) - pouch, bag from the original derivation of Thylacocephala, which referred to the large eye, then interpreted as the stomach; *acares* (Gr) - small, referring to the size of the eye in the Silurian species.

*Diagnosis:* as for the species.

*Thylacares brandonensis* sp. nov.

LSID: urn:lsid:zoobank.org:act:3A2A61CE-8133-4D43-B8A4-1FDE918E1457

*Derivatio nominis:* After the Brandon Bridge Formation, the source of the specimens.

*Holotype:* UWGM 1748, originally figured in [2], figures two and nine as UW 4001/8 and 14a.

*Paratypes:* UWGM 1749, 1750, 1767–1769, all with parts and counterparts.

*Diagnosis:* Thylacocephalan with a large bivalved shield enveloping the entire body, only eyes and distal extremities of raptorial appendages projecting beyond it. Shield in lateral view with straight dorsal margin; anterior, ventral and posterior margin of shield continuous, rounded. Optical notch very weak. Eyes relatively small, stalked. Raptorial appendages robust and stout. Trunk with up to 22 segments.

### Description of the specimens

#### General appearance

**Figure 1:**
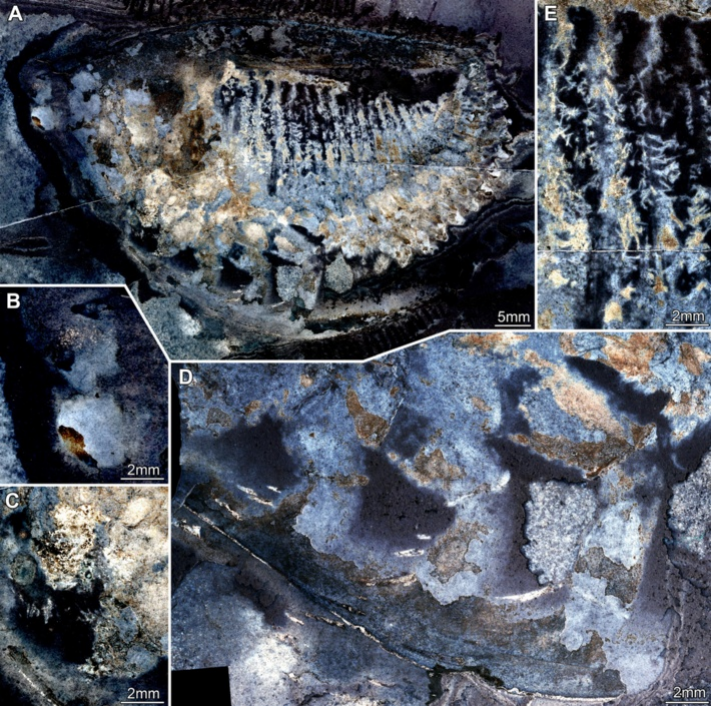
**Silurian thylacocephalan*Thylacares brandonensis*UWGM 1748.** For labeling of structures see Figure 2. **A.** Overview of part (counterpart on Figure 3) **B.** Close up of eyes. **C.** Close up of first raptorial appendage. **D.** Close up of raptorial appendages 2 and 3 (from counterpart, flipped). **E.** Close up of trunk region; small structures are fibers of calcium phosphate, probably representing the remains of muscles.

**Figure 2:**
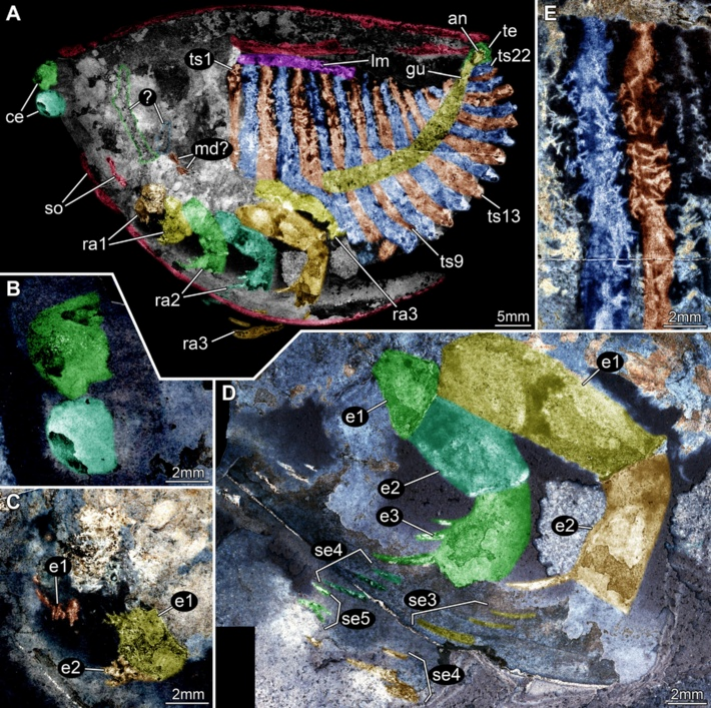
**Silurian thylacocephalan*Thylacares brandonensis*UWGM 1748.** Color-marked version of Figure 1. **A.** Overview of part (counterpart on Figure 3) **B.** Close up of eyes. **C.** Close up of first raptorial appendage. **D.** Close up of raptorial appendages 2 and 3. **E.** Close up of trunk region, probably indicating segmental muscles. Abbreviations: ? = possible appendages anterior to the raptorial appendages; an = anus; ce = compound eye; e = element; gu = gut; lm = longitudinal muscle; md? = possible mandible; ra = raptorial appendage; se = spines of element; so = shield outline; te = telson; ts = trunk segment.

**Figure 3:**
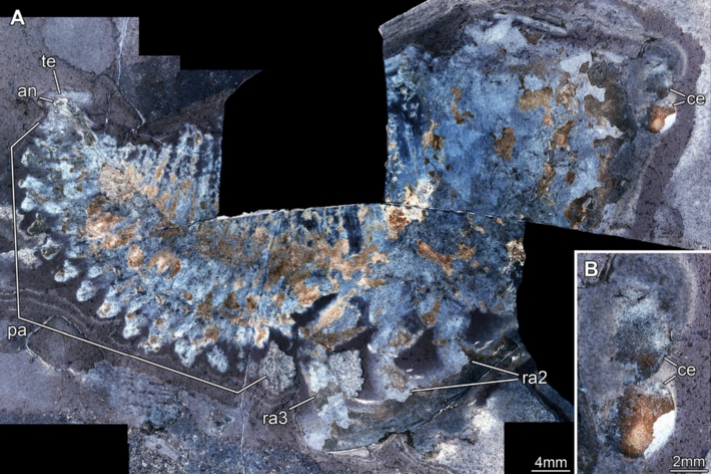
**Silurian thylacocephalan*Thylacares brandonensis*UWGM 1748.** Counterpart. **A.** Overview. **B.** Close up of eyes. Abbreviations: an = anus; ce = compound eye; pa = paddle-shaped appendages; ra = raptorial appendages; te = telson.

**Figure 4:**
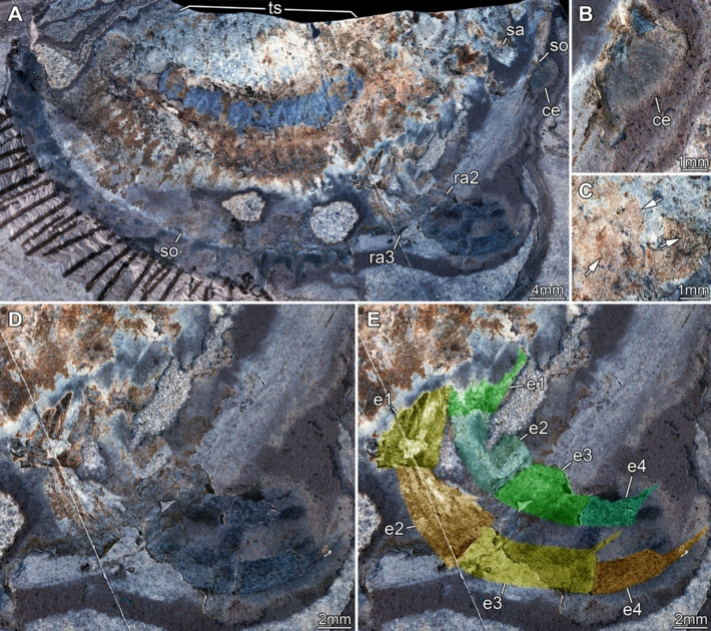
**Silurian thylacocephalan*Thylacares brandonensis*UWGM 1749. A.** Overview. **B.** Close up of eyes. **C.** Close up of supposed muscle tissue (arrows) in the trunk region. **D.** Close up of raptorial appendages 2 and 3. **E.** Color-marked version of D, indicating individual elements of the appendages. Abbreviations: ce = compound eye; e = element; ra = raptorial appendage; sa = stalk; so = shield outline; ts = trunk segments.

**Figure 5:**
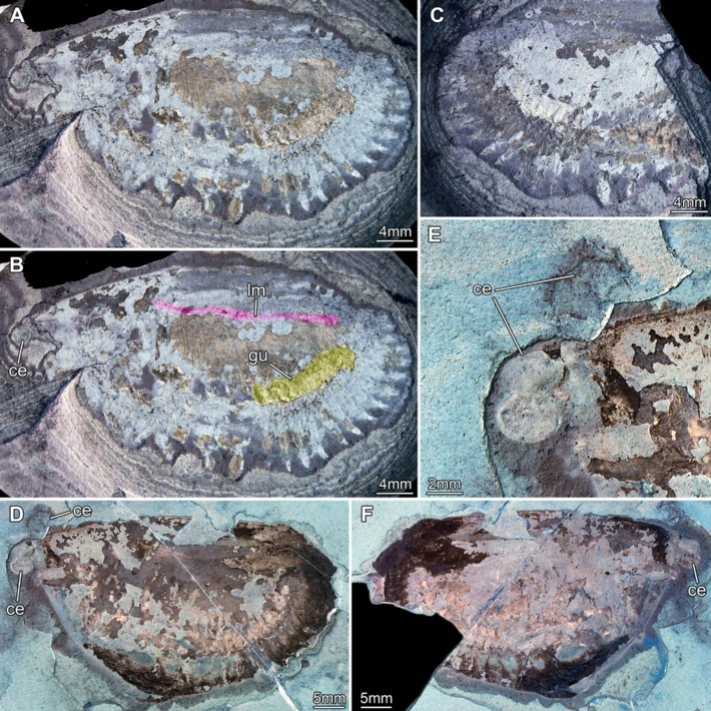
**Silurian thylacocephalan*Thylacares brandonensis*UWGM. A-C.** UWGM 1767. **A.** Overview of part. **B.** Color-marked version of A. **C.** Overview of counterpart. **D-F.** UWGM 1768. **D.** Overview of part. **E.** Close up on eye-structures. **F**. Overview of counterpart. Abbreviations: ce = compound eye; lm = longitudinal muscle; gu = gut.

**Figure 6:**
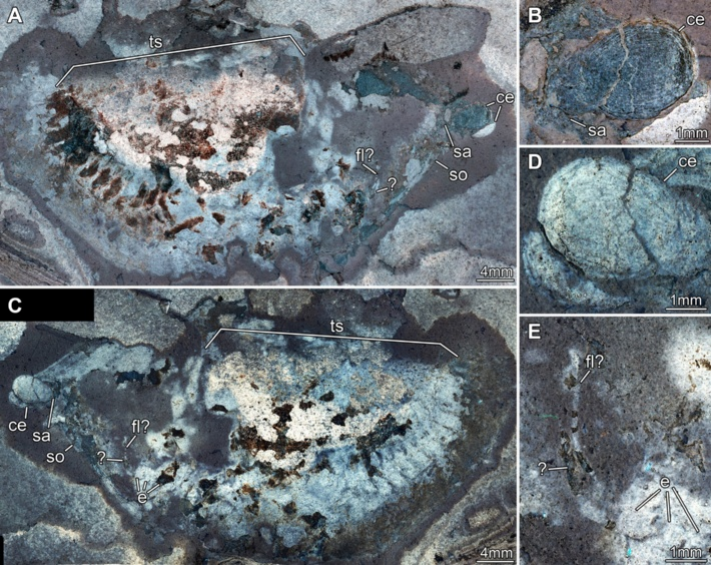
**Silurian thylacocephalan*Thylacares brandonensis*UWGM 1750. A.** Overview of part. **B.** Close-up of eyes. **C.** Overview of counterpart. **D.** Close up of eyes. **E.** Close up of possible antennula. Abbreviations: ce = compound eye; e = element; fl? = possible flagellum; sa = stalk; so = shield outline;ts = trunk segments.

Arthropod (Figures 1, 2, 3, 4, 5 and 6), length from the anterior extremity of the eyes to the distal extremity of the trunk appendages from 44 (UWGM 1767, Figure 5A) to 74 mm (UWGM 1769) (UWGM 1748 is 69 mm long, Figures 1A, 2A). The entire body is enclosed by a large bivalved shield; only the extremities of the three pairs of raptorial appendages extend beyond it (Figures 1A, 2A). The raptorial appendages appear to be borne by the head although the insertion of the third pair is close to the boundary with the trunk.

#### Shield

The attachment of the shield to the body is not clearly evident: it appears to be restricted to the anterior region of the body, i.e. the head (Figures 1A, 2A). The entire outline of the valves is smoothly rounded; the optical notch is weakly developed (Figures 1A, 2A, 3A, B). In lateral view, the posterior margin of the valves is more rounded, the anterior is slightly less so (Figure 4A).

#### Sensory structures

A pair of circular structures, presumably compound eyes, projects anteriorly from under the shield (Figures 1B, 2B, 3B, 4B, 5D-F). The eyes appear to arise from elongate stalks (Figure 6A-D). The insertion of these stalks is unknown due to preservation. Posterior of the eyes there appear to be elongate structures lying superimposed on the body, which may represent appendages (Figures 2A, 6E). Based on their anterior position, these may represent remains of the antennulae (first antennae) and/or (second) antennae, but that is unclear due to preservation. Other areas of relief in the head region might also represent appendages; the preservation is inadequate and the number of specimens too few to be sure.

#### Possible mouth parts

A pair of triangular structures lies posterior to the possible antennae (Figure 2A). Based on their position and apparently stronger sclerotisation these may represent the mandibles. Although this specimens affords a lateral view, the left and right eyes are offset suggesting that both left and right mandible appendages might both be evident, particularly if they are strongly sclerotised and likely to preserved in relief (see examples in [31],[32]. Alternatively, these structures might also represent bundles of phosphatised muscles. Further ventral to the presumed mandibles (or muscle bundles) lies the first of three pairs of raptorial appendages. The proximal part of these appendages is obscured.

#### Raptorial appendages

The first of the series of three raptorial appendages is the smallest (Figures 1C, 2C) and is probably oval-shaped in cross section. Two elements can be differentiated. The proximal element is shorter than wide and bears spines along its inner margin. The exact nature of the proximal region is unclear, as the spines are broken off. A cluster of three spines originating together is apparent distally on the proximal element. The distal element is small, providing an attachment for at least four stronger spines. They are arranged along the median edge; the proximal spines point medially, the more distal ones progressively more distally.

The second raptorial appendage is significantly larger than the first, and appears to be composed of at least five elements (Figures 1D, 2D, 4D, E); the appendage is presumably oval-shaped in cross section. The most proximal preserved element is short, about as long as wide, and lacks armature. The second element is the most elongate of the five, about 1.6 times as long as wide. The distal margin of the element is oriented oblique to the appendage axis (the outer edge of the element is about 1.6 times as long as the inner edge). The third element is only slightly shorter than the second, spinose, and angled about 45° inward. Due to the oblique joint between elements two and three, and the attitude of element three, this joint allows the appendage to function like a sub-chela. Three spines arise from the inner margin of element three, and are about three times as long as their diameter at the base. The third spine arises from the distal-median edge of the element. The two more proximal spines are about half as long as the width of the element; the third one is about twice as long but only slightly wider at its base. All three spines curve slightly inward. The position of the fourth element is evidenced only by a set of three spines, resembling those of the third element, but about 20% smaller. The fifth element is likewise only revealed by a triplet of spines, which are smaller than those of the fourth element.

The third raptorial appendage is the largest of the series, being more than 20% longer than the second one, and is presumably oval-shaped in cross section (Figures 1D, 2D, 4D, E). Four elements are known. The most proximal one is the longest and appears to correspond to the second element (or the first two elements) of the second raptorial appendage. This first element is about 2.5 times as long as wide. The distal margin is oblique; the outer edge of the element is about 1.2 times longer than the inner edge. The second element is about 70% the length of the first, spinose and angled about 45° inward. Due to the oblique joint between elements one and two, and the attitude of element two, this joint forms a functional sub-chela like that of the second raptorial appendage. A single spine arises disto-medially from element two. The spine is about as long as the width of the element, and curves slightly inward. The more distal elements are evidenced only by their spines. Element three corresponds to element four in the second raptorial appendage and strongly resembles it, differing only in its slightly larger dimensions. Element four bears three spines of which the second is the largest; it is about 1.5 times longer than the spine on element two. Proximally there is a smaller spine which is only about half the length of the second spine. Distally, part of an even smaller spine is preserved, presumably indicating the extremity of the appendage.

#### Trunk

A series of twenty-two short (in anterior-posterior dimension) bands, decreasing in height (dorsal-ventral) towards the posterior, is evident in UWGM 1748 and interpreted as the trunk (Figures 1A, 2A). The bands are probably defined mainly by internal rather than external structures, most likely the muscles within each somite (Figures 1E, 2E, 4C). The number of divisions may vary. There appear to be twenty-two in UWGM 1769 but UWGM 1767 (Figure 5A-C) preserves evidence of ~20 (based on a combination of boundaries and limbs), a lower number that may reflect the smaller size of this specimen or be a result of preservation. A narrow bundle of muscles is preserved running the length of the trunk along its dorsal margin in UWGM 1767 (Figure 5B). It is not clear whether one or both longitudinal muscles (overlapping) are preserved. Remnants of this longitudinal muscle may also be represented in UWGM 1748 (Figure 2A) running along the dorsal margin of the anterior part of the trunk; there is no evidence of a ventral longitudinal muscle. A pair of simple paddle-shaped appendages arises ventrally from each segment (Figure 3A). The position of the ventral margin of the trunk is unclear but is constrained by the trace of the gut or musculature surrounding it (which must lie within the trunk!). The position of the gut is evident in UWGM 1748 (Figure 2A), 1749 and 1767 (Figure 5B). The height (dorsal-ventral dimension) of the segment is at least 4 times the length of the appendages, which decrease in length toward the posterior. The appendages are 1.5 to 2 times as long as their preserved width. Posterior to the last segment a stout structure appears to bear the anus and is thus interpreted as the telson (Figures 2A, 3A).

### Clausocaris lithographica

#### Amended description

**Figure 7:**
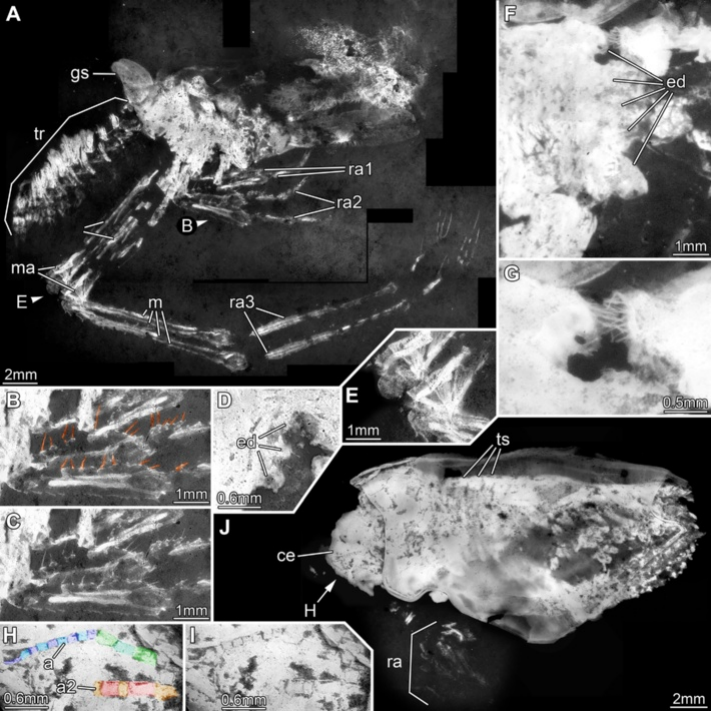
**Jurassic thylacocephalans. A-G.***Clausocaris lithographica*. **A-E.** SMNS 67901. **A.** Overview; B and E mark areas of close-ups. **B.** Close up of raptorial appendages, setae color-marked. **C**. Same as B without color-markings. **D.** Endites on raptorial appendage 2. **E.** Fan-like muscle attachment areas on raptorial appendages 3. **F-G.** SMNS 70193/1. **F.** Endites on raptorial appendage 3. **G.** Close up of F, showing opposing endites of left and right appendage. **H-J.***Mayrocaris bucculata*, SMNS 70193/5. **H.** Detail of eye with supposed antennula and antenna (color-marked). **I.** Same as H, without color-marking. **J.** Overview; arrow pointing to close-up in H (and I). Abbreviations: a = antennula; a2 = antenna; ce = compound eye; ed = endites; gs = presumed gill structure; m = muscles; ma = muscle attachment areas; ra = raptorial appendage; tr = trunk.

**Figure 8:**
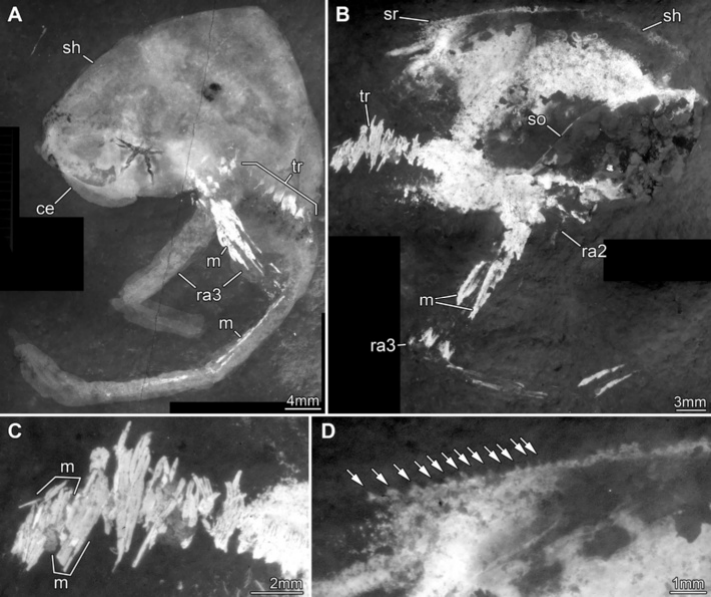
**Jurassic thylacocephalan*Clausocaris lithographica*. A.** SMNS 70193/3; note that one raptorial appendage 3 is flipped and points backwards. **B-D.** SMNS 70193/1. **B.** Overview. **C.** Close up of ventral trunk region, with distinct muscles in individual bundles. **D.** Close up of postero-dorsal rim of shield; the numerous serrations are marked by arrows. Abbreviations: ce = compound eye; m = muscles; ra = raptorial appendage; sh = shield; so = shield outline; sr = serrations; tr = trunk.

**Figure 9:**
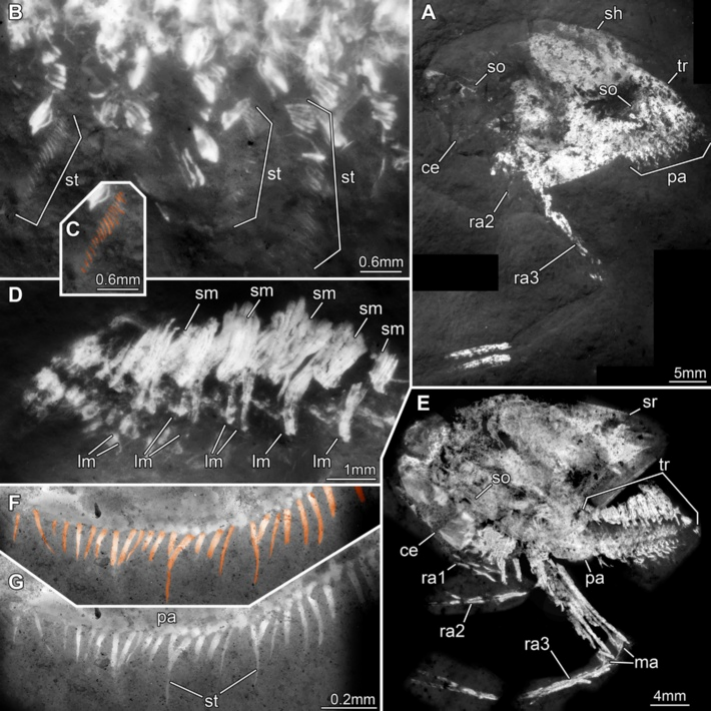
**Jurassic thylacocephalan*Clausocaris lithographica*. A-C.** SMNS 70193/2. **A.** Overview. **B.** Series of trunk appendages with preserved setae. **C**. Color-marked version of part of B, showing the setae. **D.** SMNS 67901. Close up of trunk region; note the distinct arrangement of muscles: in each segment a short muscle is followed by a long muscle which appears to extend into the appendages. **E-G.** SMNS 70193/4. **E.** Overview. **F.** Close up of paddle-shaped appendage, setae color-marked. **G.** Same as in F, without color-markings. Abbreviations: ce = compound eye; lm = long muscles; ma = muscle attachment areas; pa = paddle-shaped appendages; ra = raptorial appendage; sh = shield; sm = short muscles; so = shield outline; sr = serrations; st = setae; tr = trunk.

An extensive description of *Clausocaris lithographica* was provided by Polz [33]. Here we report new morphological details (Figures 7A-G, 8, 9) and confirm most of his observations, for example the serration of the postero-dorsal area of the shield (Figure 8D). New evidence shows that the raptorial appendages bear significantly more setae than previously observed (Figure 7B, C). The proximal region (most likely the basipod) bears enditic protrusions: at least three are present on raptorial appendage 2 (Figure 7D) and at least five on raptorial appendage 3 (Figure 7F). Each endite is equipped with at least one row of at least 8 long setae (Figure 7G). The appendages preserve long muscles (Figures 7A, 8A, B, 9A, E) with fan-like attachment areas (Figure 7E). Discrete muscle bundles are also apparent in the trunk region (Figures 8C, 9D) consisting of a bundle of shorter muscles followed by a bundle of longer ones. This pattern allows the arrangement and number of trunk segments to be determined. Displacement of the muscle bundles (Figure 9D, furthest left bundles) does not disturb this pattern. Eleven segments are evident posterior of the last raptorial appendages. Each one bears a pair of paddle-shaped appendages equipped with setae along the lateral edge (Figure 9B, F-G). The most posterior appendages are similar in structure to the preceding ones, but slightly longer (Figure 9D). Dorsally at the anterior of the trunk a pair of leaf-like structures is apparent. These may represent gills.

### Mayrocaris bucculata

#### Amended description

An extensive description of *Mayrocaris bucculata* was provided by Polz [34]. We have new evidence of two pairs of appendages lying between the two large compound eyes (preserved compressed through the eyes) (Figure 7H-J). The more anterior appendage lies slightly dorsal of the other, due to the strongly convex shape of the body in the anteriormost region. A more proximal and a distal part can be differentiated. The proximal part of the appendage consists of at least three similar elements, apparently tube-shaped and longer than wide. The distal part of the appendage appears flagellate and is comprised of at least nine elements. These also appear tube-shaped, but are significantly smaller than those of the proximal part; each element of the distal part is slightly longer than wide, the elements decreasing in width distally. It remains unclear whether additional distal elements might have been present but are not preserved. It is also uncertain whether the most proximal preserved element is the originally most proximal one, although this seems plausible. This appendage, with its anterior position, is interpreted as the antennula. The second appendage comprises five elements. These elements appear more robust than those of the first appendage, and vary in length. The two most proximal ones are about twice as long as wide. The third element is only one third of the length of the preceding one. The fourth one is longer again, about twice as long as element 3. Element 5 appears to be similar in length to element 3, but may be incomplete. It remains unclear whether further distal elements were present but are not preserved. This appendage is interpreted as the antenna.

Further posterior under the shield dorso-ventral bands are apparent. Such structures have been interpreted as gills [34]. Yet, here these structures seem more likely to represent the more anterior trunk segments.

## Discussion

### Preservation of details

**Figure 10:**
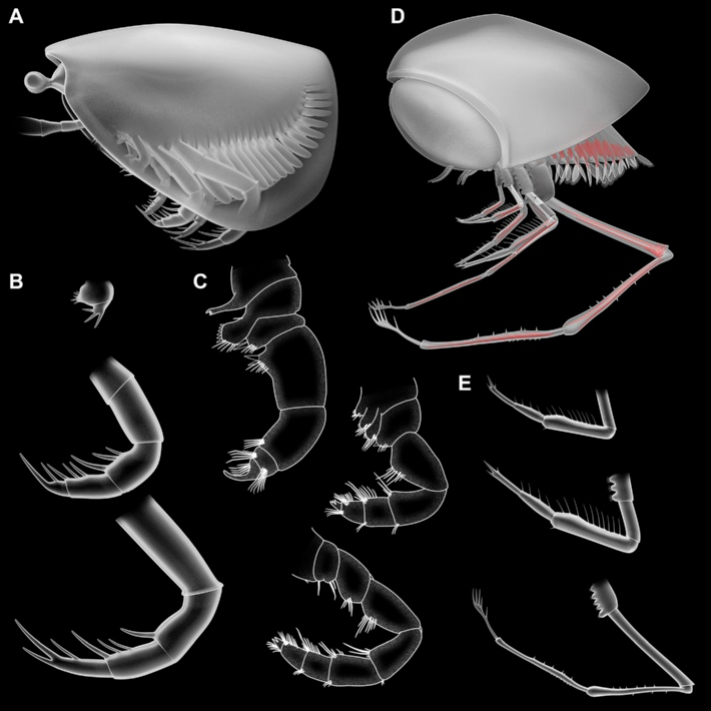
**Reconstructions of thylacocephalan morphology and comparison to remiped morphology. A-B.***Thylacares brandonensis*. **A.** Overview. **B.** Raptorial appendages. **C.** Appendages of the remiped “*Speleonectes*” *epilimnius* based on [48]. **D-E.***Clausocaris lithographica*. **D.** Overview. **E.** Raptorial appendages.

The relatively common preservation of muscles in specimens of *Clausocaris lithographica* (Figure 10D) is remarkable. The long muscles (Figures 7A, 8A-B, 9A, D), and their fan-shaped attachment (Figure 7D), are unusual. Even more so are the tightly arranged muscles in the trunk, which also preserve a distinct pattern (Figure 8C). These structures were observed by Polz [33], who interpreted them as parts of the trunk appendages. The distinction between trunk and appendages is evidenced by the setae on the latter (Figure 9C, F, G). The appendages are represented mainly by muscle and setae indicating that their cuticle was rather weakly sclerotised and lost through decay. Muscles are also preserved in each trunk segment of *Thylacares brandonensis* (Figures 2E, 4C) and help to define their boundaries and identify the segment count.

### Assignment of *Thylacares brandonensis* to Thylacocephala

*Thylacares brandonensis* shows many characters typical of thylacocephalans, but also some features that are unusual for the group (Figure 10A). The large bivalved shield lacks the characteristic well developed optical notch (Figure 3A, B), a feature that was used to argue that some Cambrian taxa should be assigned to Thylacocephala [4]. Yet, other thylacocephalans appear to lack a pronounced optical notch [6]. This observation emphasizes that the shield morphology of bivalved arthropods is not a reliable guide to their affinity (e.g. [35]).

The eyes of *T. brandonensis* are also unusual in their small size and apparent stalked nature (Figure 6A-D). The large compound eyes of other species of Thylacocephala are generally regarded as sessile [11]. Yet the eyes of many other thylacocephalans are unknown and may also have been small and stalked.

*T. brandonensis* bears three pairs of sub-chelate appendages (Figure 2A) that resemble those of other thylacocephalans in general structure and size, i.e. the first one is the smallest, the third the largest. Yet they are significantly shorter than those described in most other species (compare Figure 10B and Figure 10E).

The trunk of *T. brandonensis* is very similar to that of other representatives of Thylacocephala, although it is comprised of more segments.

The differences between this Silurian form and other thylacocephalans can be interpreted as plesiomorphic. A marked optical notch in some later thylacocephalans may be a derived feature, yet shield structures are highly likely to be convergent, and the plesiomorphic condition is difficult to infer. From a functional point of view the evolution of a notch is likely coupled to the reduction of the eye stalks and enlargement of the eyes in some forms. The long raptorial appendages of some later thylacocephalans, which contrast with the short limbs in the Silurian form, also appear to be a derived character, as other younger forms retain relatively short raptorial appendages [6].

### The absence of gills

The presence of eight gills has been advocated as an important character of Thylacocephala (e.g. [12],[17]). While it is difficult to judge whether a structure in a fossil is a gill [36], the exquisite preservation in some of the fossils from La Voulte allows virtually no other interpretation [12],[17].

We have observed no features in *T. brandonensis* that resemble gills. *T. brandonensis* may represent a sister species to all other thylacocephalans, in which case the absence of gills might be plesiomorphic; alternatively gills may have been lost through decay.

The features interpreted as gills in *M. bucculata*[34] may simply represent the upper preserved part of the anterior trunk segments; they are in a similar position to these segments in *T. brandonensis.* Eight poorly preserved gills have also been reported in *C. lithographica*[33], but they are not evident in the specimens we investigated. Thus, while some thylacocephalans appear to have eight sets of (supposed) gills, it is not clear whether this is a diagnostic character of the group.

### Assignment to Eucrustacea

Most authors have considered Thylacocephala as an ingroup of Eucrustacea, yet unequivocal evidence for this assignment has been lacking. Lange et al. [37] noted that the presence of two pairs of antennae in *Thylacocephalus cymolopos* from the Upper Cretaceous of Lebanon supports the assignment of thylacocephalans to crustaceans. There is also evidence of two pairs of antennae in *Thylocaris brandonensis*. Although the second appendage is called an ‘antenna’ in Eucrustacea, it is antenniform only in Eumalacostraca. In other eucrustaceans it is used mainly for locomotion, and sometimes resembles the mandible but never the antennula (see discussion in [38]). Thus the morphology of the second antenna varies in different eucrustaceans and this character must be used with caution in determining affinity.

The morphology of the appendages of Thylacocephala appears to be highly derived, which makes comparison with other arthropods difficult. One new observation reported here supports a eucrustacean affinity. The proximal region of the raptorial appendages of *Clausocaris lithographica* bears up to five enditic projections with rows of setae. Median enditic armature is widespread among Euarthropoda. Slight elevations in early chelicerates bear just a single strong spine accompanied by two smaller spines. Similar arrangements are found in early crustaceans. In labrophoran crustaceans the endites are more strongly pronounced and bear more complex armature (see e.g. [38]). Several strong endites with setose armature are developed in entomostracan eucrustaceans and in at least some appendages of malacostracan eucrustaceans. Hence the presence of up to five pronounced endites with numerous setae on the raptorial appendages of Thylacocephala supports a eucrustacean affinity.

### Systematic position: earlier ideas

Since their recognition as a separate group [16],[30] thylacocephalans have been assigned to a range of eucrustacean groups including stomatopods, decapods and cirripedes, and a superficial resemblance to Remipedia has also been noted [3],[39].

The comparison with stomatopods (see discussion in [3]and [40] was based on the shape of the shield of some Cretaceous species, which in lateral aspect resembles that of certain stomatopod larvae. The appendage morphology, however, is incompatible with stomatopods. Although the raptorial appendages of stomatopods are described as sub-chelate they differ strongly from those of thylacocephalans in overall structure. Most important of these differences is the double flexure that results in a Z-shape in stomatopods, whereas the raptorial appendages of thylacocephalans are only “folded” once, and do not close fully. The distal movable finger in stomatopods is formed by a single element, while it comprises four or five in thylacocephalans. There are five pairs of sub-chelate appendages in stomatopods, the second of the series being the largest (at least in extant forms), while in Thylacocephala the last of three pairs is the largest.

The arrangement of tagmata in thylacocephalans, and especially in *Thylacares brandonensis*, argues against a malacostracan affinity, including stomatopods. The trunk of up to 22 undifferentiated segments strongly differs from that of Malacostraca, which is consistently differentiated into a thorax of eight segments and a pleon of six (sometimes five in Eumalacostraca) or seven (Phyllocarida). The arrangement of tagmata in Thylacocephala also rules out a decapod affinity, a suggestion prompted by the similarity of the gills in certain thylacocephalan species to those in decapods [17]; the nature of the gills in fossil arthropods is difficult to infer without evidence of the ultrastructure of the surface epithelium [36].

The long trunk does not support a close affinity between Thylacocephala and Cirripedia (as pointed out by [41],[42]); the trunk of cirripedes includes only six segments. Like cirripedes, thylacocephalans possess pits on their shield [42], which may represent a dorsal organ. While the special arrangement of these pits in the so-called lattice organ has been argued to be an autapomorphy of Euthecostraca (which also includes cirripedes: [43]) such pits are widespread among crustaceans and even other euarthropods [44].

### Systematic affinities: sistergroup to Remipedia?

The new details reported here prompt a reconsideration of a possible affinity of thylacocephalans to remipedes supporting suggestions by Schram [39]). Remipedes possess three pairs of sub-chelate appendages (Figure 10C): the posterior two head appendages (maxillula and maxilla) and the first trunk appendage (maxilliped).

Three pairs of limbs are present anterior to the raptorial appendages in *Clausocaris lithographica*, namely antennula, antenna and mandibles [19]. Our observations reveal at least two pairs of anterior appendages in *Mayrocaris bucculata* (most likely representing antennula and antenna, similar to strucures observed in *Thylacocephalus cymolopos*[37]) and possibly three in *Thylacares brandonensis* (antennula, antenna and mandibles). Thylacocephala in general appear to bear at least three pairs of appendages anterior to the first raptorial one.

The segmental affiliation of the three raptorial appendages in Thylacocephala is uncertain. They have been interpreted as belonging to the anterior trunk segments (see summary in [19]), but their position in the specimens described here makes this unlikely. The raptorial appendages are broad proximally and apparently too robust to attach to the short trunk segments, except perhaps the most anterior ones. Both their position and size indicates that at least some of the raptorial appendages belong to the posterior divisions of the head.

Thus the three pairs of raptorial appendages in Thylacocephala could represent maxillula, maxilla and trunk limb one (maxilliped), or maxilla and trunk limbs one and two. Additional material is required to resolve this question but a positional homology (homotopy) between the raptorial appendages of Thylacocephala and Remipedia is at least plausible.

Morphological similarities between the raptorial appendages in the two groups strengthen this assumption. The proximal part of the appendages (probably a basipod) bears setose endites in both. More importantly, three or more distal elements form the functional finger of the subchela in thylacocephalans as well as in remipedes (Figure 10B, C, E). This is an unusual character state, as the functional finger of other subchelae in crustaceans comprises only the most distal element (e.g. in mantis shrimps and gammarids), or the distalmost two (e.g. in slipper lobsters).

The multisegmented and relatively undifferentiated trunk in *Thylacares brandonensis*, which bears more than twenty appendages, is unusual among eucrustaceans. Apart from the modern branchiopod ingroups *Polyartemia* and Phyllopoda, which, unlike thylacocephalans, possess phyllopodous appendages (i.e. limbs differentiated into a basipod with median endites, a reduced endopod, a paddle-shaped exopod and lateral epipods; e.g. [45]), only Remipedia show such a high number of trunk segments. The eucrustacean trunk is usually differentiated into at least two tagmata: thorax and pleon in malacostracans and thorax and abdomen in entomostracans [46], in contrast to the trunk in thylacocephalans and Remipedia, where there is only one tagma. The specialisation of the posterior head appendages and anteriormost trunk appendage as sub-chelate raptorial appendages with setose endites and a finger made up of the three or more distal elements represents a potential synapomorphy of Thylacocephala and Remipedia. This, together with the multisegmented trunk, suggests a sister-group relationship.

Additional material is necessary to determine whether the three raptorial appendages in Thylacocephala are homologous in position with maxillula, maxilla and maxilliped and to test the possibility of a sister group relationship with Remipedia. Such evidence is a prerequisite for a rigorous phylogenetic analysis.

### Functional morphology and 3D modelling of Thylacocephala

Previous authors (e.g., [4],[20]) have considered the thylacocephalans to be nectic or necto-benthic predators. The new evidence presented here allows us to refine these interpretations.

Thylacocephalans are usually reconstructed with their raptorial appendages pointing forward, i.e., more or less in the axial plane of the body. However, our new reconstruction (Figure 10A, D) shows that such an arrangement is unlikely. Given the narrow ventral gape, which has been reconstructed in different species [4], and the relatively large size of the appendages, only certain positions are possible. The proximal podomeres with their endites and armature must have interacted with the opposing appendage of the pair and therefore cannot face directly forward, but at most antero-medially. When the valves are closed, the largest (third) appendages would occupy almost the entire width of the ventral gape; there is no space for the two other pairs of raptorial appendages to lie inside or outside the valves. This problem is overcome by rotating the proximal podomeres about 45° abaxially to accommodate all three appendages within the narrow ventral gape in an anterior-posterior sequence.

In remipedes the appendages are also held in an abaxial orientation, at an even higher angle than in thylacocephalans. Mantis shrimps, although not closely related, provide a further functional comparison. They accommodate four pairs of sub-chelate appendages which are also rotated away from the axis. The arrangement of the raptorial appendages in mantis shrimps, near parallel to the axial plane, is achieved by the presence of an additional joint, resulting in a z-shape.

The attitude of thylacocephalan raptorial appendages reconstructed here is supported by several specimens that preserve them directed posteriorly (Figures 7E, F, 8A). Preservation of appendages projecting forwards in some specimens and backwards in others is less likely if the appendages are held in an orientation parallel to the axial plane of the body.

The appendages are reconstructed here forming a kind of basket. Such an arrangement would have facilitated predation. In the absence of a second, strongly flexed joint, such as that in mantis shrimps and some great-appendage arthropods [47], the raptorial appendages of thylacocephalans could not extend forward. More likely, the crustacean swam forward using the trunk appendages and trapped prey or at least grabbed food items with the raptorial appendages.

The paddle-shaped trunk appendages identified here in *Clausocaris lithographica* are compatible with a swimming function. They are equipped with setae enlarging the surface, and powered by well-developed muscles. The last pair of trunk appendages, which are more elongate than those lying anterior to them, may have functioned as a steering device, in a manner similar to the uropods of eumalacostracans and the furca of phyllocarid and entomostracan eucrustaceans.

## Conclusions

1. The Silurian thylacocephalan *Thylacares brandonensis*, described here for the first time, appears less derived than many of the later representatives of the group.

2. Features “typical” for many thylacocephalans, such as a marked optical notch on the shield, sessile hypertrophied eyes and extremely elongate raptorial appendages, as well as a relatively short trunk, evolved after the early Paleozoic.

3. The new evidence reinforces the assignment of Thylacocephala to Eucrustacea.

4. Previous hypotheses of the position of Thylacocephala within Eucrustacea (to Stomatopoda, Decapoda or Thecostraca) are incompatible with the new information reported here.

5. This new information suggests that a sistergroup relationship between Thylacocephala and Remipedia merits further testing.

## Competing interests

The authors declare that they have no competing interests.

## Authors’ contributions

DGM and JK initiated research on the Waukesha fauna and locality, and conducted sedimentologic, biostratigraphic, sequence stratigraphic and ∂C^13^ isotope studies to document the temporal and depositional controls on this unique biota; CH, DEGB and JTH documented these specimens, JTH created the reconstructions, CH, DEGB and JTH wrote the paper with input from the other authors, all of whom read and approved the final manuscript.
